# Airport Malaria Cluster in Certified Malaria-Free Country, Libya, 2024

**DOI:** 10.3201/eid3202.251508

**Published:** 2026-02

**Authors:** Ahmed M. Alarbi, Ahmed B. Elhaddad, Nafesa M. Almehdawi, Walid K. Saadawi, Hanan Aqeehal, Mohamed Elalem

**Affiliations:** Mediterranean and Black Sea Programme in Intervention Epidemiology Training, European Centre for Disease Prevention and Control, Stockholm, Sweden (A.M Alarbi); National Center for Disease Control, Tripoli, Libya (A.M. Alarbi, W.K. Saadawi, H. Aqeehal, M. Elalem); University of Benghazi, Benghazi, Libya (A.B. Elhaddad); Benghazi Medical Center, Benghazi (A.B. Elhaddad, N.M. Almehdawi)

**Keywords:** malaria, Plasmodium falciparum, parasites, vector-borne infections, airports, disease outbreaks, public health surveillance, Anopheles, Culex, Libya

## Abstract

In November 2024, an autochthonous cluster of 4 *Plasmodium falciparum* cases (1 fatal) was identified near Benina International Airport, Benghazi, Libya. Epidemiologic and entomologic investigation ruled out local transmission, pointing to airport malaria as the cause and highlighting the vulnerability of malaria-free regions to imported vectors.

Libya, which was certified malaria-free by the World Health Organization in 1973, faces continuous risk for malaria reintroduction from population movements in malaria-endemic regions (*1*). Although imported cases are occasionally reported, local transmission has not been documented in eastern Libya for >50 years.

On November 28, 2024, the Libya National Centre for Disease Control was notified of a fatal case of *Plasmodium falciparum* malaria in a 63-year-old resident of Benghazi, Libya, who had no history of travel. Within 48 hours, infection was confirmed in 3 of his children (ages 12, 16, and 23). This familial cluster was located ≈450 meters from Benina International Airport, prompting an investigation to determine the outbreak’s source. This investigation was considered a public health response to an urgent event by the Libya National Center for Disease Control and, as such, was not subject to institutional review board approval. Oral informed consent was obtained from the family members for interviews and testing.

Our investigation defined a case as malaria-like symptoms in a resident near the airport during from mid-November through mid-December 2024 (Figure 1). Active case finding through local healthcare and community outreach identified 20 other suspected patients; all tested negative. All 4 confirmed case-patients, belonging to a single family of 8, were laboratory-confirmed as positive for *P. falciparum*. None had traveled internationally or received blood transfusions. We formulated 2 primary hypotheses: local transmission from indigenous *Anopheles* mosquitoes infected by human carrier or airport malaria from an infected *Anopheles* mosquito imported by aircraft.

**Figure 1:**
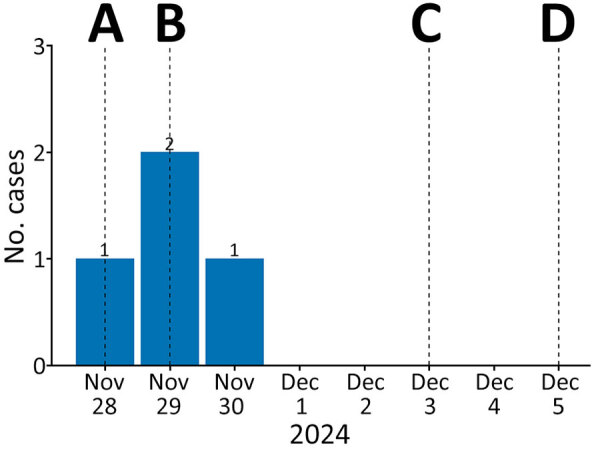
Confirmed malaria cases by symptom onset and public health response timeline in study of airport malaria cluster in certified malaria-free country, Benghazi, Libya, November–December 2024. A) Index case death notified; B) investigation and case finding initiated; C) no *Anopheles* mosquito vectors confirmed in traps; D) public health recommendations issued.

To evaluate those hypotheses, we conducted entomological surveillance during November 29–December 2, 2024, deploying National Centre for Disease Control light traps around the family’s residence and within the airport perimeter (Figure 2). We collected 8 mosquitoes, all of which were identified as *Culex pipiens*. We found no *Anopheles* mosquitoes, a finding consistent with recent national surveillance data, which documented certain *Anopheles* species in specific ecologic niches but confirmed their general absence in coastal urban areas such as Benghazi (H. Aqeehal, unpub. data). The absence of competent local vectors enabled us to eliminate local transmission, making airport malaria the most plausible explanation (*2*).

**Figure 2:**
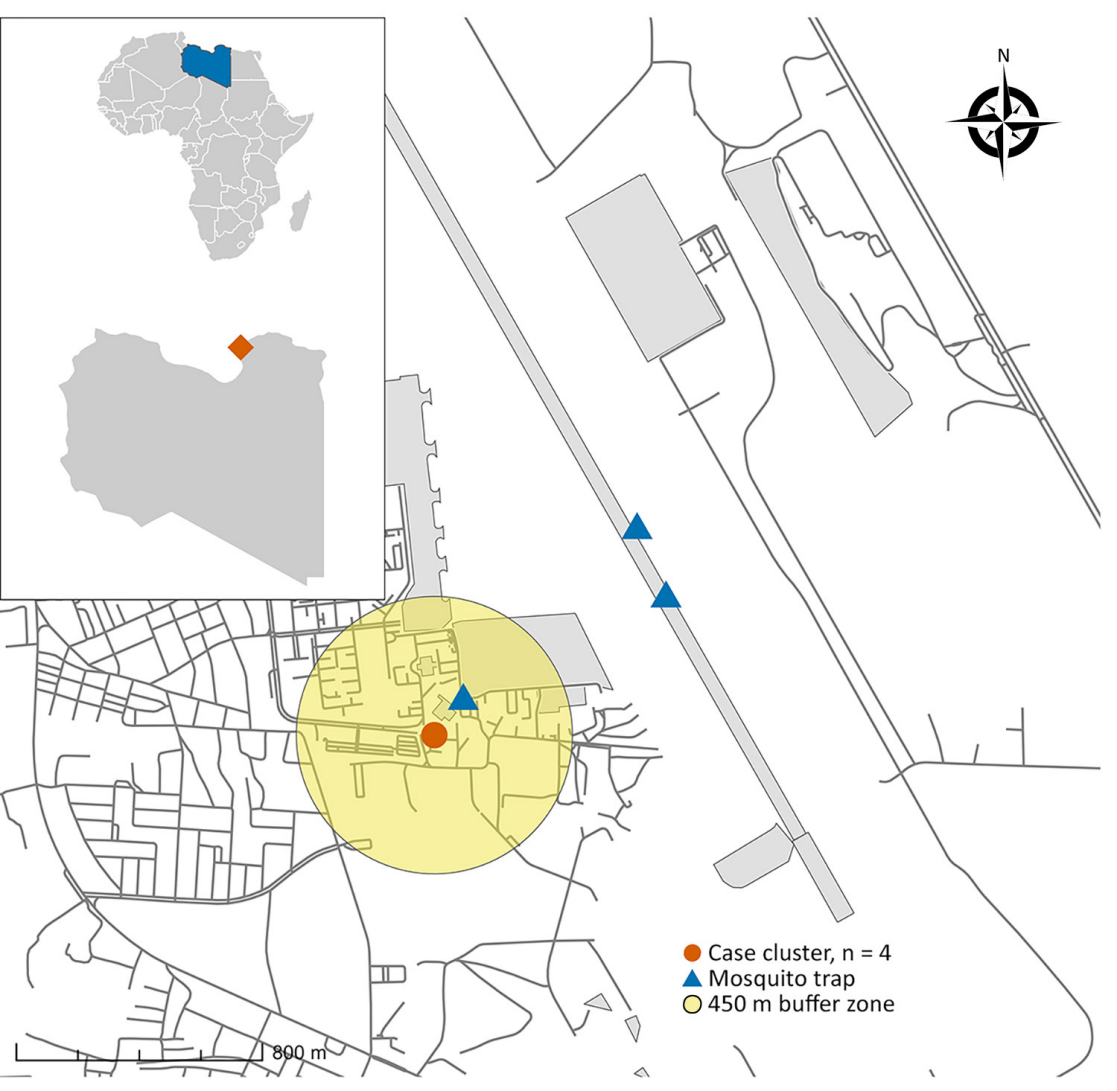
Location of airport malaria cluster in certified malaria-free country, Benghazi, Libya, November 2024. The map displays the location of the case cluster (n = 4) and mosquito traps relative to the Benina International Airport runway. The yellow shaded area indicates a 450-meter buffer zone from the cluster center. Inset maps show the location of Libya in Africa and Benghazi within Libya.

This investigation concluded that an infected anopheline mosquito was likely imported by aircraft, probably within cargo given the absence of recent passenger flights from malaria-endemic areas, and subsequently infected members of a family living nearby. Although importation of vectors overland through migrant routes is a theoretical possibility (*3*,*4*), the acute, geographically tight cluster in a nonmigrant family points strongly toward a point-source introduction at the airport.

This event is a critical reminder that malaria-free status does not eliminate risk, because points of entry are permeable frontiers for vectorborne diseases (*5*,*6*). Our findings prompted immediate recommendations to Libya health authorities to strengthen entomologic surveillance at points of entry and rigorously enforce aircraft and cargo disinsection protocols in accordance with the World Health Organization’s International Health Regulations (2005) (*7*). We also emphasize the need for clinicians near airports to consider malaria in patients with fever, regardless of travel history. The vulnerability of nonendemic regions requires constant vigilance to prevent the reestablishment of malaria.
